# Effect of Lengthy Root Canal Therapy Sessions on Temporomandibular Joint and Masticatory Muscles

**DOI:** 10.5681/joddd.2010.024

**Published:** 2010-09-16

**Authors:** Safoora Sahebi, Fariborz Moazami, Masoomeh Afsa, Mohammad Reza Nabavi Zade

**Affiliations:** ^1^ Assistant Professor, Department of Endodontics, Faculty of Dentistry, Shiraz University of Medical Sciences, Shiraz, Iran; ^2^ Associate Professor, Department of Endodontics, Faculty of Dentistry, Shiraz University of Medical Sciences, Shiraz, Iran; ^3^ Post-graduate Student, Department of Oral Radiology, Faculty of Dentistry, Shiraz University of Medical Sciences, Shiraz, Iran

**Keywords:** Long duration, mouth opening, root canal therapy, temporomandibular dysfunction

## Abstract

**Background and aims:**

Trauma is one of the major factors associated with temporomandibular joint disorders (TMD). These disorders result from macro-trauma or micro-trauma. Macro-trauma might be iatrogenic; for example, from intuba-tion procedures, third molar extraction procedures, and lengthy dental appointments. The aim of this study was to evaluate the effect of lengthy root canal therapy (more than 2 hours) on TMJ and its supporting structures.

**Materials and methods:**

Eighty patients whose root canal therapy session lasted more than 2 hours were examined for the status of TMJ and masticatory muscles. After one week the second part of the examination was carried out for TMJ problems and pain and tenderness levels of masticatory muscles. Data was analyzed using Wilcoxon statistical test.

**Results:**

Women showed more pain compared to men. There was a significant increase in pain in the external acoustic meatus examination one week after root canal therapy. Patients who were treated for their posterior teeth suffered more pain than those who were treated for the anteriors and premolars. Other aspects of the examination were not affected significantly by lengthy root canal therapy.

**Conclusion:**

Lengthy dental treatments can harm TMJ and masticatory muscles and wide opening of the mouth during such appointments can worsen the situation. Therefore, it is wise to break the appointment into shorter intervals and let the patients rest during treatment to close their mouth to prevent iatrogenic damage to TMJ.

## Introduction

The masticatory system is the functional unit of the body primarily responsible for chewing, speaking and swallowing. This system is made up of bones, joints, ligaments, teeth, and muscles. Functional disturbances of the masticatory system are identified by the term temporomandibular disorders (TMD). This term does not suggest merely problems that are confined to the joints but includes all the disturbances associated with the function of the masticatory system. TMD is identified as a major cause of non-odontogenic pain in the orofacial region and is considered to be a sub-classification of musculoskeletal disorders.^1^

Some studies have reported a high prevalence of functional disorders in the masticatory system. These studies report that on average 40-60% of the population have at least one detectable sign associated with TMD.^2 , 3^ A range of symptoms may be linked to TMD; pain, predominantly in the masticatory muscles and/or jaw joint, is the most common symptom. Probable indications of TMD include limited movement or locking of the jaw, radiating pain in the face, neck, or shoulder muscles, painful clicking or grating sounds in the jaw joint when opening or closing the mouth, and a sudden change in occlusal status. Symptoms such as headaches, earaches, dizziness, and hearing problems may sometimes be associated with TMD.^4^

A review of literature reveals five major factors associated with TMD: (1) occlusal condition; (2) trauma; (3) emotional stress; (4) deep pain input; and (5) parafunctional activities. Trauma can be divided into two general types: (1): macro-trauma and (2) micro-trauma. Micro-trauma refers to any small force that is repeatedly applied to the structures over a long period of time. Activities such as bruxism or clenching can lead to micro-trauma. Macro-trauma is considered any sudden force to the joint that can result in structural alterations. Macro-trauma may be indirect, referring to the injury inflicted on the TMJ secondary to a sudden force; it may be direct, such as a blow to the chin. This type might be iatrogenic. Whenever the jaw is overextended, elongation of the ligaments can occur. A few common examples of iatrogenic trauma are intubation procedures, third molar extraction procedures, and lengthy dental appointments. In fact, any lengthy wide opening of the mouth (e.g. a yawn) has the potential of elongating the disc ligaments.^1^

Although it has been claimed that TMD is a result of manipulations related to routine dental examinations, oral endotracheal intubation for general anesthesia, and lengthy dental procedures, including lengthy wide opening of the mouth, stretching or forcing the mouth to open for restorative and orthodontic treatments, tooth extraction or orthognathic surgeries, there is no scientific evidence that common or routine dental or medical procedures lead to TMD.^5^

Although it has been reported that there is little causal relationship between third molar removal and TMJ injury,^6^ some authors suggest that mouth opening for a long period of time and the exertion of a variable force on the mandible during some surgeries can traumatize one or both TMJs.^7^

It has been reported that one of the causes involved in TMD is lengthy opening of the mouth;^5^therefore, we decided to evaluate the effect of lengthy root canal therapy sessions (more than 2 hours) on TMJ and its supporting structures.

## Materials and methods

Eighty patients with no significant systemic problems and no severe TMJ disorders were randomly selected from those patients who were referred to the Department of Endodontics at Shiraz Faculty of Dentistry. A consent form was signed by each patient following clear explanation of the steps of the study. The patients underwent root canal treatment which lasted for more than 2 hours; the patients could not close their mouth during the procedure because rubber dam was applied.

Before commencing the treatment, a demographic form was filled for each patient to record general information including age, gender, systemic disorders the patient suffered from and the tooth to be treated. In addition, the history of prior trauma and TMD, evidence of bruxism or clenching, missing teeth, and existing prosthetic appliances were recorded.

Preoperative examination of the TMJ and masticatory muscles was carried out for each patient. These examinations included palpation of masseter, temporalis, medial and lateral pterygoid, sternocleidomastoid and trapezius muscles to detect any tenderness or spasm. Maximum jaw opening without pain or discomfort was measured as inter-incisal distance (the distance between the incisal edges of upper and lower central incisors). Both TMJs were examined for jaw sounds, such as clicks or crepitus. In addition, periauricular palpation and intraacoustic meatus examinations were carried out to rule out pain in the region.

One week after treatment, the second part of the form was filled which included all parts of the first examination. At this time, patients were asked if they experienced TMJ problems such as pain, masticatory muscles tenderness, jaw sounds, and locked jaw. Again maximum jaw opening was measured for each patient to detect limitation of mouth opening after root canal therapy.

Finally, pre- and post-operative data was compared statistically using Wilcoxon test, as the outcome values were not normally distributed.

## Results

Statistical analysis showed that one week postoperatively external acoustic meatus pain increased significantly (P=0.007). The results also revealed that pain was reported more frequently in patients who were treated for their molar teeth than those who were treated for their anterior or premolar teeth. The results also showed that after treatment women experienced more pain compared to men.

The other aspects of examination were not affected significantly by the lengthy root canal therapy session.

## Discussion

Root canal therapy, especially for multi-rooted teeth, by an inexperienced student is usually a lengthy treatment. The presence of rubber dam in the mouth does not let patients close their mouth during treatment. Our results revealed that wide opening of the mouth during lengthy endodontic treatment sessions (more than 2 hours) can give rise to signs and symptoms associated with TMD.

In this study female subjects complained of more pain postoperatively than males. With the exception of some sporadic series, most studies have reported that TMD is common in women than in men, with female-to-male ratios between 1.75:1 and 3:1,^8^consistent with our findings. Biological, cultural, hormonal or environmental factors acting alone or in combination may be responsible for the reported association between TMD and female gender.^9^ Women have smaller mouths and their maximum jaw opening is smaller relative to men, resulting in more discomfort after wide opening of the mouth for a long time.

As mentioned previously, wide opening of the mouth for a long time can be a major trauma to TMJ with its consequences.^1^ Although we could not find any study specifically evaluating the effect of lengthy root canal therapy on TMJ, there are studies reporting that lengthy surgical procedures such as third molar tooth removal can give rise to TMD in patients undergoing such treatments.^6 , 7^ During such procedures, muscles of mastication and articular ligaments are stretched for an extended period of time, which can cause muscle spasm, pain and discomfort during mouth opening and chewing, limiting mouth opening.

In root canal therapy for posterior teeth patients need to open their mouth wider and for a longer period of time. In this situation, more force is applied to the muscles and ligaments, resulting in more TMJ pain and dysfunction after treatment.

Procedures that need lengthy mouth opening are not confined to dental appointments.

One situation in which the patient undergoes lengthy mouth opening is endotracheal intubation because of general anesthesia during surgical procedures. Endotracheal intubation has been reported as a risk factor for TMD, in which symptoms may result from forces applied with the laryngoscope, or may be related to the duration in which TMJ structures are stretched.^10^

## Conclusion

Lengthy dental treatment sessions and wide opening of the mouth during such procedures can harm temporomandibular joints or worsen the TMD which is already present. Therefore, it is prudent to break treatment session into shorter appointments and also let the patient rest and relax the muscles during treatment to prevent iatrogenic damage to TMJs.
